# Phenotypic spectrum and molecular characteristics of variants of uncertain significance in copy number variations associated with fetal cardiac malformations: a case series of 9 patients

**DOI:** 10.3389/fped.2026.1696089

**Published:** 2026-03-10

**Authors:** Jun Yin, Qingsong Wang, Ting Lan, Huimin Ou, Xiaoqin Zhou, Lingli Zhong, Hongmei Cao, Le Zhang, Yan Luo

**Affiliations:** 1 Department of Ultrasound, Pediatrics, Cardiology, Obstetrics and Gynecology, Anorectal Surgery, West China Hospital Sichuan University Jintang Hospital, Jintang First People’s Hospital, Chengdu, Sichuan, China; 2 Pediatric Cardiology Center, Sichuan Provincial Women’s and Children’s Hospital/The Affiliated Women’s and Children’s Hospital of Chengdu Medical College, Chengdu, Sichuan, China

**Keywords:** chromosomal microarray analysis, congenital heart malformations, genetic counseling, genotype–phenotype correlation, postnatal follow-up, prenatal diagnosis, variant of uncertain significance

## Abstract

**Background:**

Copy number variants of uncertain significance (VUS-CNVs) pose significant challenges in prenatal counseling for congenital heart malformations (CHMs). Their clinical relevance remains poorly defined, particularly in the absence of postnatal validation.

**Objective:**

To characterize the phenotypic and molecular features of VUS-CNVs in CHM fetuses and evaluate their clinical implications.

**Methods:**

From a cohort of 644 fetuses with CHMs undergoing chromosomal microarray analysis (CMA), we identified 9 VUS-CNV cases. All reportable CNVs (≥200 kb or in known pathogenic regions such as 22q11.21) were validated by TaqMan qPCR. Detailed prenatal echocardiography, trio CMA (*n* = 7), and postnatal follow-up were integrated for comprehensive assessment.

**Results:**

VUS-CNVs were identified in 9 of 644 (1.40%) CMA-tested fetuses with CHMs. These included 3 in isolated CHM, 1 in complex CHM, 3 in CHM with extracardiac structural anomalies, and 2 in CHM with isolated soft markers. VUS-CNVs in non-isolated cases were larger (median: 2.5 Mb vs. 0.72 Mb in isolated CHM) and encompassed more OMIM-listed genes (mean: 3.8 vs. 1.5 in isolated CHM) than those in isolated CHM. Trio analysis reclassified 3 of 7 tested VUS-CNVs (2 as likely benign, 1 as likely pathogenic). Six pregnancies continued to term—3 with isolated and 3 with non-isolated CHM—and all live-born infants showed no major postnatal abnormalities. The three terminations involved non-isolated phenotypes; decisions were primarily driven by structural anomaly severity, though the VUS amplified prognostic uncertainty.

**Conclusion:**

Although VUS-CNVs are uncommon in fetuses with CHMs, they occur more frequently in non-isolated phenotypes and carry the potential for future reclassification—posing significant challenges in prenatal genetic counseling. These two aspects underscore the necessity of trio-based genomic testing, cautious interpretation of results, and longitudinal follow-up. Given the limited sample size of this study, no causal relationship between VUS-CNVs and clinical phenotypes can be inferred; however, our findings support a risk-stratified management framework that integrates detailed fetal phenotyping, trio analysis, and postnatal follow-up to enable accurate variant interpretation and informed decision-making.

## 1 Introduction

Congenital heart malformations (CHMs) are among the most prevalent structural birth defects, with an estimated incidence of 0.8%–1.2%, and represent a leading cause of neonatal morbidity and mortality (1). Advances in prenatal imaging—particularly the widespread adoption of fetal echocardiography—have substantially improved the detection of cardiac anomalies *in utero*. Nevertheless, the precise genetic etiology of many CHMs remains incompletely understood. Accumulating evidence indicates that copy number variations (CNVs), defined as submicroscopic deletions or duplications of genomic DNA, contribute significantly to the pathogenesis of CHMs (2).

Chromosomal microarray analysis (CMA) has become a cornerstone of prenatal genomic evaluation in the context of fetal congenital heart malformations. However, not all detected copy number variants (CNVs) can be definitively classified according to current guidelines, and those designated as variants of uncertain significance (VUS-CNVs) present a significant clinical challenge (3). Although the objective pathogenic risk of most VUS-CNVs is low, their inherent ambiguity often provokes substantial parental anxiety and may influence reproductive decisions—including pregnancy termination—even in the absence of clear evidence linking the variant to the observed phenotype (4). This concern is tempered by follow-up data showing that many prenatal VUS-CNVs are inherited from phenotypically normal parents or are later reclassified as benign upon additional evidence (5), thereby underscoring the critical need for cautious interpretation and avoidance of overattribution during prenatal counseling.

We present a detailed analysis of nine fetuses with CHMs and VUS-CNVs identified within a cohort of 644 CMA-tested pregnancies.By integrating high-resolution prenatal phenotyping, orthogonal validation of all reported CNVs, trio-based inheritance data (available for seven cases), and postnatal outcomes, we aim to delineate the genomic and clinical features associated with these variants. Importantly, we refrain from inferring causality; rather, our goal is to provide a descriptive, evidence-informed framework to support nuanced and balanced genetic counseling in the face of diagnostic uncertainty.

## 2 Methods

### 2.1 Study design and participants

This study employed a retrospective design, collecting data from 3,386 pregnant women who underwent amniocentesis between January 2020 and August 2022 at Sichuan Provincial Maternal and Child Health Care Hospital. Among these cases, 697 fetuses were diagnosed with CHMs via fetal echocardiography. CHMs were defined as structural cardiac anomalies detectable by ultrasound, including septal defects, conotruncal malformations, left/right ventricular outflow tract obstructions, and complex lesions (e.g., heterotaxy, single-ventricle physiology). Cases with isolated soft markers (e.g., mild pyelectasis, choroid plexus cysts, echogenic bowel, nuchal translucency ≥3.5 mm) without structural cardiac defects were excluded from the main cohort; however, if these soft markers co-occurred with confirmed CHMs, the cases were included. Of the 697 CHM cases, 644 underwent CMA, leading to the identification of nine VUS-CNVs.

### 2.2 Prenatal phenotypic assessment

Fetal echocardiography was performed using a GE Voluson E8 system equipped with a 4–8 MHz probe. Standard views—including four-chamber, left/right ventricular outflow tracts, great artery short-axis, aortic arch, ductal arch, and inferior vena cava long-axis—were used to assess cardiac anatomy, shunt flow, vessel dimensions, and valve function. All diagnoses were independently confirmed by two certified prenatal diagnostic specialists. Detailed anomaly scans (18–24 weeks' gestation) documented: (1) Specific CHM type and severity; (2) Presence of extracardiac structural anomalies (e.g., renal agenesis, neural tube defects); (3) Ultrasound soft markers associated with aneuploidy or genomic disorders.Soft markers were defined as isolated sonographic findings associated with increased aneuploidy risk but not constituting structural anomalies, including absent/hypoplastic nasal bone, echogenic intracardiac foci, mild ventriculomegaly (<10 mm), or intrahepatic punctate echogenic foci.

### 2.3 Chromosome karyotype analysis

Amniocentesis was performed under ultrasound guidance, and approximately 10–20 mL of amniotic fluid was collected. After incubation at 37 °C, cells were separated by centrifugation and cultured in bottles for seven days. The culture medium was changed after seven days, and cell growth was observed 24 h later. Cells were then harvested, subjected to hypotonic treatment, fixed, spread onto slides, and stained using G-banding according to the International System for Human Cytogenetic Nomenclature (ISCN 2024).

### 2.4 Chromosomal microarray analysis

Genomic DNA was extracted from amniotic fluid samples and analyzed using the Agilent SurePrint G3 Human CGH + SNP 8 × 60 K microarray platform, which provides an effective resolution of 50 kb for deletions and 100 kb for duplications. Hybridization and scanning were performed on the Agilent SureScan Dx scanner, and data were analyzed using Agilent CytoGenomics software.

According to institutional reporting standards and ACMG/ClinGen technical guidelines, all CNVs meeting either criterion were validated by orthogonal TaqMan qPCR prior to clinical disclosure:Size ≥200 kb, or Size <200 kb but overlapping a known genomic disorder region.Regions of homozygosity (ROH) > 10 Mb were reported separately and not classified as copy-number loss.

Final interpretation of CNVs followed ACMG 2020 guidelines, ISCN nomenclature, and expert consensus, integrating evidence from DECIPHER, ClinVar, DGV, OMIM, and PubMed databases. Regions of homozygosity (ROH) > 10 Mb were reported separately and not classified as copy-number loss.

### 2.5 Trio analysis and VUS reclassification

Parental samples were offered for CMA or targeted qPCR upon VUS identification. Trio analysis was completed in 7 of 9 cases. Inheritance patterns guided reclassification per ClinGen recommendations: *de novo* variants involving dosage-sensitive genes were upgraded to “likely pathogenic,” while variants inherited from phenotypically normal parents were downgraded to “likely benign.” Reclassification occurred at the time of parental result integration.

### 2.6 Follow-up

Pregnancy outcomes were ascertained through medical records and, when necessary, telephone follow-up with referring obstetricians or families. To validate prenatal cardiac diagnoses, postnatal echocardiography was performed in all live-born infants within 72 h of delivery by pediatric cardiologists blinded to the prenatal CMA results.

## 3 Results

### 3.1 Genomic and phenotypic characteristics of VUS-CNVs

Between January 2020 and August 2022, among 3,386 pregnancies undergoing amniocentesis at Sichuan Provincial Maternal and Child Health Care Hospital, Affiliated Women and Children's Hospital of Chengdu Medical College, 697 fetuses were diagnosed with congenital heart malformations (CHMs) by fetal echocardiography. Chromosomal microarray analysis (CMA) was performed in 644 of these cases (92.4%); the remaining 53 cases did not undergo CMA due to parental refusal (*n* = 41) or insufficient DNA sample (*n* = 12).Among the 644 CMA-tested cases, clinically reportable copy-number variants (CNVs) were identified in 43 (6.68%), including 34 pathogenic or likely pathogenic CNVs (5.28%) and 9 variants of uncertain significance (VUS-CNVs, 1.40%). The remaining 601 cases (93.3%) showed no reportable CNVs—that is, they either had a normal CMA result or carried benign/likely benign variants that were not considered clinically relevant. In these 9 CHM cases harboring VUS-CNVs, the variant sizes ranged from 0.37 Mb to 26.0 Mb. The VUS-CNVs exhibited marked heterogeneity in genomic content and associated prenatal phenotypes, spanning a spectrum from isolated cardiac anomalies to complex presentations involving extracardiac structural malformations or ultrasound soft markers. The complete cohort flow and genetic findings are summarized in Table 1.

**Table 1:** Cohort flow and genetic findings from 3,386 pregnancies to 9 VUS-CNV cases.

Step	Description	n (%) or n
Total cohort	Pregnancies undergoing amniocentesis (Jan 2020–Aug 2022)	3,386
↓	Fetal echocardiography diagnosed with congenital heart malformation (CHM)	697
↓	Underwent chromosomal microarray analysis (CMA)	644 (92.4%)
• Excluded from CMA	Parental refusal	41
• Excluded from CMA	Insufficient DNA sample	12
↓	CMA results (*n* = 644)	
• Pathogenic/likely pathogenic CNVs	Clinically reportable, diagnostic yield	34 (5.28%)
• Variants of uncertain significance (VUS-CNVs)	Primary focus of this study	9 (1.40%)
• No reportable CNVs	Normal or benign/likely benign variants	601 (93.3%)
↓	Phenotypic subgroups among 9 VUS-CNV cases	
• Isolated (simple) CHM	Mild cardiac defects only	3
• Complex CHM	Multiple intracardiac anomalies (e.g., TOF)	1
• CHM+extracardiac structural anomalies	e.g., ventriculomegaly, intestinal dilation	3
• CHM+soft markers only	e.g., absent nasal bone, echogenic foci	2

No reportable CNVs=normal CMA or variants classified as benign/likely benign per ACMG guidelines.

To systematically describe this phenotypic diversity, the 9 VUS-CNV cases were classified into four subgroups based on prenatal ultrasound findings, as follows:(1) Complex CHM (*n* = 1, Case 9): A large 21.2 Mb deletion at 2q31.1q32.3 was observed in a fetus with tetralogy of Fallot (TOF); (2) Isolated (simple) CHM (*n* = 3, Cases 2, 6, 8): Smaller VUS segments (0.37–1.07 Mb) were associated with mild cardiac findings, including small ventricular septal defects and mild tricuspid regurgitation;(3) CHM with structural extracardiac anomalies (*n* = 3, Cases 4, 5, 7): Larger VUS (2.13–26.0 Mb) co-occurred with severe multisystem involvement, including bilateral lateral ventricular widening, fetal intestinal dilation, and clubfoot; (4) CHM with soft markers only (*n* = 2, Cases 1, 3): VUS sizes were 2.47 Mb and 2.55 Mb, respectively, accompanied by isolated soft markers such as absent nasal bone and intrahepatic punctate echogenic foci.

VUS-CNVs in fetuses with structural extracardiac anomalies were larger (median: 21.2 Mb; range: 2.13–26.0 Mb) and encompassed more OMIM-listed genes (mean: 5.2) compared to those in isolated CHM (median: 0.72 Mb; range: 0.37–1.07 Mb; mean: 1.5). The two VUS-CNVs in the soft markers group showed intermediate size (2.47 and 2.55 Mb) and gene load (2.5 ± 0.7). Given the small number of cases in each subgroup (*n* ≤ 3), these differences are reported descriptively and should not be interpreted as statistically significant.These findings suggest that larger VUS-CNVs and higher gene burden tend to co-occur with more complex prenatal phenotypes, although no statistical inference can be made due to the small number of cases.

To contextualize the genomic features of VUS-CNVs, we compared their size distribution across CHM subtypes with that of the 34 pathogenic or likely pathogenic CNVs (Table 2). In isolated CHM, VUS-CNVs were uniformly small (0.37–1.07 Mb), with two of three (66.7%) < 1 Mb—smaller than the majority of pathogenic CNVs in this subgroup (only 18.5% < 1 Mb). Conversely, in non-isolated phenotypes (complex CHM or extracardiac anomalies), VUS-CNVs tended to be large: all three cases with extracardiac anomalies had variants ≥2.13 Mb (including one 26.0 Mb ROH), and the single complex CHM case carried a 21.2 Mb deletion. This contrasts with pathogenic CNVs in similar phenotypes, where sizes were more evenly distributed across categories (e.g., 50% of pathogenic CNVs in extracardiac anomaly group were 1–4 Mb). Notably, the two VUS-CNVs in the “soft markers only” subgroup were both in the 1–4 Mb range (2.47 and 2.55 Mb), a size interval also commonly observed among pathogenic CNVs.

**Table 2:** Comparison of CNV size distribution between pathogenic/likely pathogenic CNVs and VUS-CNVs across CHM subtypes.

CHM Subtype	Group	n	<1 Mb n (%)	1–4 Mb n (%)	≥5 Mb n (%)
Isolated (simple) CHM	Pathogenic CNVs	27	5 (18.5)	13 (48.1)	9 (33.3)
	VUS-CNVs	3	2 (66.7)	1 (33.3)	0 (0.0)
Complex CHM	Pathogenic CNVs	5	1 (20.0)	2 (40.0)	2 (40.0)
	VUS-CNVs	1	0 (0.0)	0 (0.0)	1 (100.0)^a^
CHM with extracardiac structural anomalies	Pathogenic CNVs	2	0 (0.0)	1 (50.0)	1 (50.0)
	VUS-CNVs	3	0 (0.0)	1 (33.3)	2 (66.7)^b^
CHM with soft markers only	Pathogenic CNVs	0	—	—	—
	VUS-CNVs	2	0 (0.0)	2 (100.0)	0 (0.0)

^a^ Case 9: 21.2 Mb deletion at 2q31.1q32.3.

^b^ Includes one 26.0 Mb ROH (Case 4) and one 2.13 Mb deletion (Case 7).

### 3.2 Individual case descriptions

The genomic characteristics and associated phenotypes of the nine VUS-CNVs are summarized in Table 3 and described individually as follows:

**Table 3:** VUS analysis of 9 cases of heart malformations in fetuses.

Case	Age (years)	Gestational Age (weeks)	Ultrasound Findings	CMA Result	VUS	Length (Mb)	Suspected Pathogenic Genes
1	25	24 + 4	Fetal nasal bone absent, tricuspid regurgitation	Normal	14q22.1q22.2 Heterozygous Deletion	2.47Mb	*BMP4,PTX2,SIX6,GCH1,TMEM260*
2	31	24 + 3	Mild tricuspid regurgitation, left ventricular focal strong echo?	Normal	17q11.2 Duplication	0.49Mb	*NF1*
3	38	23 + 2	Fetal intrahepatic punctate strong echo, possible calcification, tricuspid regurgitation, strong echo in both ventricles	Normal	2p16.1p15 Duplication	2.55Mb	*BCL11A,PAPOLG,REL*
4	28	24 + 5	Bilateral lateral ventricular widening, tricuspid regurgitation	Normal	12q15q22 Heterozygous Deletion	26Mb	*OTOGL,BBS10,ALX1*
5	27	30 + 5	Fetal intestinal dilation, bilateral renal pelvis separation, persistent left superior vena cava, tricuspid regurgitation, left ventricular focal strong echo	Not Done	18q23 Heterozygous Deletion	2.13Mb	*TXNL4A*
6	32	24 + 2	Fetal ventricular septal defect, tricuspid regurgitation, persistent left superior vena cava	Not Done	22q11.21 Duplication	0.37Mb	*HNF1B*
7	28	24 + 5	One twin of a dichorionic diamniotic pregnancy with absent venous duct, clubfoot, single umbilical artery, persistent left superior vena cava	Normal	5q22.1q22.2 Duplication	2.28Mb	-
8	27	26 + 3	Fetal ventricular septal defect 2mm	Normal	13q12.2q12.3 Duplication	1.07Mb	*PAN3,FLT1,POMP,SLC46A3*
9	29	18 + 6	One twin of a pregnancy with Tetralogy of Fallot (VSD, overriding aorta, pulmonary stenosis)	Normal	2q31.1q32.3 Heterozygous Deletion	21.2Mb	

Case 1: A 25-year-old woman at 24 + 4 weeks of gestation underwent prenatal ultrasound, which revealed tricuspid regurgitation and an absent nasal bone. Chromosomal karyotype analysis yielded a negative result. CMA detected a heterozygous deletion of approximately 2.47 Mb in the *14q22.1q22.2* region, classified as a VUS-CNVs. This deletion encompasses 15 protein-coding genes, including *BMP4* (OMIM: 112262). *BMP4* is associated with haploinsufficiency, although the evidence is currently insufficient (Haploinsufficiency score: 2). *BMP4* is linked to Oculofaciocardiodental Syndrome 6 (OMIM: 607932) and Cleft Lip/Palate (OMIM: 600625). The clinical phenotypes associated with *BMP4* are heterogeneous, primarily manifesting as ocular abnormalities (microphthalmia/anophthalmia), dysmorphic facial features, genitourinary system malformations, polydactyly/syndactyly, and developmental delay (PMID: 21340693, 18252212, 31053785). No similar variants were reported in the Database of Genomic Variants (DGV). One case with a VUS-CNVs classification was found in the DECIPHER database (ID: 257464, *de novo*, corneal opacity, polydactyly, intellectual disability). One pathogenic case was reported in the ClinVar database (RCV000511921, *de novo*, anxiety, global developmental delay, dysmorphic facial features).

Case 2: A 31-year-old woman at 24 + 3 weeks of gestation underwent prenatal ultrasound, which indicated mild tricuspid regurgitation and a possible echogenic focus in the left ventricle. Chromosomal karyotype analysis yielded a negative result. CMA detected a duplication of approximately 500 kb (0.49 Mb) in the *17q11.2* region, classified as a VUS-CNVs. No similar variants were reported in the DGV population database. This segment includes 6 protein-coding genes and the 5'UTR and exons 1–5 of the *NF1* gene (NM_001042492.3, total 58 exons). The *NF1* gene is a well-established haploinsufficient gene listed in the ClinGen database (HI = 3), but it is not triplosensitive (TS = 0). *NF1* (OMIM: 613113) deletions are associated with Neurofibromatosis Type 1 (OMIM: 162200), an autosomal dominant disorder. Clinical manifestations include cardiac abnormalities, scoliosis, café-au-lait spots, dysmorphic facial features, hypertelorism, intellectual disability, and neurofibromas. The precise physical location of this duplication is unknown, and it remains uncertain whether it leads to abnormal expression of the *NF1* gene.

Case 3: A 38-year-old woman at 23 + 2 weeks of gestation underwent prenatal ultrasound, which showed echogenic foci within the liver (possible calcification), tricuspid regurgitation, and echogenic foci in both ventricles. Chromosomal karyotype analysis yielded a negative result. CMA detected a duplication of approximately 2.55 Mb in the *2p16.1p15* region, classified as a VUS-CNVs. This segment includes 14 protein-coding genes such as *BCL11A*, *PAPOLG*, and *REL*, among which 3 are OMIM disease-associated genes. No similar variants were reported in the DGV population database. This region largely overlaps with the known *2p15p16.1* region (including *BCL11A*) (hg19 chr2:59139200-62488871), which is recognized as a haploinsufficient region but with insufficient evidence for triplosensitivity (ClinGen Haploinsufficiency score: 3; Triplosensitivity Score: 1). Patients with duplications in this region have been reported to exhibit autism or mild global developmental delay (PMID: 22726847, 26278498). Pathogenic/likely pathogenic case reports exist in both the DECIPHER (patients 366385, 331051, 257648, 265052, 1570) and ClinVar (RCV000626531) databases.

Case 4: A 28-year-old woman at 24 + 5 weeks of gestation underwent prenatal ultrasound, which revealed mild ventriculomegaly and tricuspid regurgitation. Chromosomal karyotype analysis yielded a negative result. CMA analysis did not identify any clinically significant copy number variants (CNVs), but detected a 26 Mb heterozygous region of loss of heterozygosity (ROH) in the *12q15q22* region, classified as a VUS-CNVs. This ROH region does not contain known imprinting control regions associated with pathogenic genes. However, it increases the risk for autosomal recessive disorders within the ROH interval, such as Deafness (OMIM: 614944, gene: *OTOGL*), Bardet-Biedl Syndrome Type 10 (OMIM: 615987, gene: BBS10), and Frontonasal Dysplasia (OMIM: 613456, gene: ALX1).

Case 5: A 27-year-old woman at 30 + 5 weeks of gestation underwent prenatal ultrasound, which indicated dilated bowel, bilateral renal pelvis separation, persistent left superior vena cava (PLSVC), tricuspid regurgitation, and an echogenic focus in the left ventricle. Chromosomal karyotype analysis was not performed. CMA detected a heterozygous deletion of approximately 2.13 Mb in the *18q23* region, classified as a VUS-CNVs. This region contains 11 protein-coding genes and does not include any known dosage-sensitive genes or regions. No similar variants were reported in the DGV population database. Multiple pathogenic reports were found in the ClinVar database (e.g., RCV000053908.5). Pathogenic reports were also found in the DECIPHER database (patients 385579, 286111, 385452, 411551), where patients exhibited non-specific symptoms such as short stature and choanal atresia. Additionally, several VUS-CNVs reports exist in both databases (e.g., DECIPHER patients 292936, 2343, 438849).

Case 6: A 32-year-old woman at 24 + 2 weeks of gestation underwent prenatal ultrasound, which revealed a ventricular septal defect (VSD), tricuspid regurgitation, and persistent left superior vena cava (PLSVC). Chromosomal karyotype analysis was not performed. CMA detected a duplication of 381 kb (0.37 Mb) in the *22q11.21* region, classified as a VUS-CNVs. Reports in the DECIPHER and ClinVar databases include pathogenic, likely pathogenic, VUS-CNVs, and likely benign classifications for this region. Patients' clinical phenotypes include small for gestational age, dysmorphic facial features, cardiovascular morphological abnormalities, and mild intellectual disability. Multiple reports exist in the DGV population database. This duplication involves 9 protein-coding genes, and the ClinGen database does not list any triplosensitivity effects for these genes/regions.

Case 7: A 28-year-old woman at 24 + 5 weeks of gestation (dichorionic diamniotic twins) underwent prenatal ultrasound for one twin, which revealed absence of the ductus venosus, clubfoot, single umbilical artery, and persistent left superior vena cava (PLSVC). Chromosomal karyotype analysis yielded a negative result. CMA detected a duplication of 2.28 Mb in the *5q22.1q22.2* region, classified as a VUS-CNVs. No pathogenic reports were found in the DECIPHER or ClinVar databases. No reports were found in the DGV population database. One likely benign report exists in the ClinVar database. This duplication involves 12 protein-coding genes. The ClinGen database has no reports of triplosensitivity effects for this region.

Case 8: A 27-year-old woman at 26 + 3 weeks of gestation underwent prenatal ultrasound, which revealed a 2 mm ventricular septal defect (VSD). Chromosomal karyotype analysis yielded a negative result. CMA detected a duplication of 1.07 Mb in the *13q12.2q12.3* region, classified as a VUS-CNVs. Overlapping regions (approximately 50%–80%) in the DECIPHER and ClinVar databases have several reports classified as VUS-CNVs or likely benign. No similar variants were found in the general population database DGV. This region contains 4 OMIM genes (*PAN3, FLT1, POMP, SLC46A3*). The ClinGen database did not report triplosensitivity effects for the duplicated genes.

Case 9: A 29-year-old woman at 18 + 6 weeks of gestation (dichorionic diamniotic twins) underwent prenatal ultrasound for one twin, which revealed Tetralogy of Fallot (TOF) (VSD, overriding aorta, pulmonary stenosis). Chromosomal karyotype analysis yielded a negative result. CMA analysis did not identify any clinically significant chromosomal copy number variants, but detected a heterozygous loss of heterozygosity (ROH) of approximately 21.2 Mb in the *2q31.1q32.3* region, classified as a VUS-CNVs. This region does not contain known pathogenic imprinting genes. However, it contains multiple recessive disease genes, which may increase the risk for associated autosomal recessive disorders, such as short stature associated with the *AGPS* gene (OMIM: 603051), Muscular Dystrophy Type 6 associated with the *MAP3K20* gene (OMIM: 6094790), Mandibular Hypoplasia, Deafness, and Progeroid Syndrome associated with the *MTX2* gene (OMIM: 608555), and Deafness Type 59 associated with the *PJVK* gene (OMIM: 610219).

### 3.3 Pregnancy outcomes, postnatal validation, and VUS reclassification

All nine pregnancies with VUS-CNVs were followed to outcome. Six resulted in live births following detailed genetic counseling—two with isolated CHM and four with CHM plus structural extracardiac anomalies. Postnatal echocardiography was performed in all six infants within the first week of life: cardiac findings were consistent with prenatal diagnoses in five cases, while one infant (Case 2) showed resolution of an isolated echogenic intracardiac focus, a well-recognized benign variant. During short-term follow-up (median age 4 months), all live-born infants remained free of major complications, overt dysmorphic features, or neurodevelopmental delays.

The remaining three pregnancies were electively terminated after multidisciplinary counseling. Parental decisions reflected the interplay between fetal phenotype severity and VUS-related uncertainty: (1) In Case 1 (14q22.1q22.2 del), termination was primarily driven by parental anxiety regarding potential neurocognitive risks, despite reassurance about the variant's uncertain clinical significance; (2) In Case 5 (18q23 del), multiple structural anomalies—including cardiac defect, intestinal dilation, and renal hypoplasia—led to termination, with the VUS contributing to perceived genomic risk; (3) In Case 9 (2q31.1q32.3 del), severe tetralogy of Fallot was the dominant factor, though the large VUS intensified concerns about long-term prognosis.

As of the study endpoint (August 2025), three of the nine VUS-CNVs were reclassified through our institutional protocol for periodic CMA re-evaluation, which integrates updated evidence from ClinGen, DECIPHER, and OMIM with trio-based inheritance analysis (performed in 7/9 cases). Specifically: Two variants inherited from healthy parents were downgraded to likely benign; One *de novo* variant involving a dosage-sensitive cardiac gene was upgraded to likely pathogenic.The remaining six variants (including two without parental samples) retained VUS classification due to insufficient evidence. Notably, this reclassification increased the overall diagnostic yield in our cohort of 644 fetuses from 34/644 (5.28%) to 35/644 (5.43%).

## 4 Discussion

### 4.1 Cardiac malformations and genetic testing

CHMs are among the most common fetal developmental anomalies, with genetic factors playing a pivotal role in their pathogenesis. Current prenatal genetic testing primarily relies on chromosomal karyotyping and CMA. CMA enables molecular karyotyping through various array platforms and offers greater sensitivity than conventional karyotyping, allowing for the detection of genomic microdeletions and microduplications. It has been widely adopted in clinical practice for the evaluation of unexplained chromosomal abnormalities and is now established as a first-tier cytogenetic diagnostic tool for individuals with developmental delay, autism spectrum disorders, and multiple congenital anomalies (6). In parallel, CMA has become the recommended genetic test for pregnancies with fetal structural abnormalities on ultrasound or a history of adverse obstetric outcomes. Owing to its superior sensitivity and faster turnaround time, CMA detects clinically significant submicroscopic copy number variants (CNVs) in approximately 5.2% of cases (7, 8). A large cohort study by Srebniak et al. demonstrated that the implementation of CMA increased the detection rate of pathogenic CNVs from 1.7% to 4.3% in pregnancies with ultrasound anomalies (9). Similarly, a study focusing on fetal CHMs reported, in line with prior findings, a 5.2% incremental yield in the detection of pathogenic CNVs smaller than 10 Mb compared to conventional karyotyping (10). Thus, CMA not only provides critical molecular diagnostic information in the context of fetal CHMs but also contributes valuable genetic data for research and counseling. Furthermore, across the entire genome, CMA can simultaneously detect chromosomal numerical and structural abnormalities, as well as low-level mosaicism, making it a comprehensive and indispensable tool in the clinical evaluation of congenital structural anomalies, including CHMs (11).

CMA not only complements traditional karyotyping in diagnosing fetal CHMs but also identifies the underlying genetic causes of birth defects and specific gene variants associated with CHMs by detecting genomic deletions or duplications, known as copy number variants (CNVs). Emerging evidence indicates that CNVs play a crucial role in the pathogenesis of fetal CHMs, with an increasing variety of CNVs affecting cardiac-related genes being identified. A large cohort study revealed that the *22q11.2* deletion syndrome is the most common microdeletion syndrome, occurring in approximately 1 in 5,950 live births and accounting for 0.5%–1.9% of all fetal CHMs (12). Previous studies have established the roles of *GATA4, NKX2*, and *TBX5* in monogenic cardiac malformations (13), while *BMP4* and *CRELD1* have also been implicated in the development of fetal CHMs (14).

Currently, a subset of detected CNVs have uncertain clinical significance and cannot be accurately classified, termed variants of uncertain significance (VUS-CNVs), with the potential for future reclassification as pathogenic with accumulating evidence (15). These VUS-CNVs include variants that: (1) reside in regions lacking protein-coding or functionally important genes but exceed the laboratory's reporting threshold; (2) encompass a few genes with unknown dosage sensitivity; or (3) have been reported in the literature with conflicting conclusions, leaving their definitive clinical significance unresolved. In this study, among 644 fetuses with CHMs who underwent CMA, 53 cases (8.23%) harbored CNVs, including 44 pathogenic CNVs and 9 VUS-CNVs. Among the 9 VUS-CNVs, database interrogation revealed 3 involving genes associated with fetal CHMs (*NF1, HNF1B, MAP3K20*). Five VUS-CNVs contained multiple protein-coding genes linked to conditions such as distinctive facial features, orofacial malformations, genitourinary anomalies, and intellectual disability (6 16, 17). One VUS-CNVs showed no detectable gene expression in existing databases. Of the 9 VUS-CNVs, 3 were associated with fetal CHMs. Follow-up of pregnancy outcomes for these 3 cases revealed 2 healthy live births (both <4 years old at manuscript writing) and 1 pregnancy termination, suggesting potential pathogenicity. These findings provide new data for comprehensive genetic counseling, although further studies are needed to confirm this observation.

### 4.2 Phenotypic spectrum and molecular characteristics of VUS-CNVs

This study demonstrates differences in VUS-CNVs detection rates across CHM subtypes: lowest in isolated malformations (0.54%, 3/553), 1.54% in complex malformations (1/65), 12.5% in cases with extracardiac structural anomalies (3/24), and 100% in cases associated with ultrasound soft markers (2/2), indicating heterogeneous genetic underpinnings. Isolated CHMs may have lower genetic heterogeneity, potentially arising from the cumulative effect of common variants, whereas complex or non-isolated CHMs are more frequently associated with rare CNVs and single-gene variants, reflecting a more complex genetic background. This finding aligns with previous studies: CMA is more likely to detect submicroscopic abnormalities, including VUS-CNVs, in non-isolated CHMs and ventricular septal defects (17); all cardiac defects with additional anomalies carried CNVs, with a VUS-CNVs detection rate of 23.5% (6) (compared to 19.23% in this study); and the detection rate of single-gene variants in complex CHMs is significantly higher than in isolated CHMs (36.4% vs. lower rates), with higher overall diagnostic rates in multisystem involvement (OR = 2.41) (20, 21), further supporting a strong association with genetic abnormalities (22). Notably, this study also found that larger VUS-CNVs fragments are more prevalent in patients with extracardiac structural anomalies or complex CHMs and are accompanied by higher genomic burden, suggesting a potential correlation with more severe clinical phenotypes. These findings indicate that VUS-CNVs not only exhibit phenotypic specificity in detection frequency but may also influence clinical severity through their size and gene content, although their definitive pathogenicity requires further functional studies and long-term follow-up.

Previous studies have identified VUS-CNVs associated with complex CHMs in regions such as *3q29, 5q22.1-22.2*, and *9p22*, which may be linked to cardiac developmental gene regulatory networks (23). In this study, one VUS-CNVs related to complex CHM involved a heterozygous deletion in *2q31.1q32.3*, while the remaining 7 VUS-CNVs associated with isolated or non-isolated CHMs involved microdeletions or microduplications on chromosomes 2, 5, 12, 13, 14, 17, 18, and 22. Database analysis using OMIM, ClinGen, and ClinVar revealed that 88.9% (8/9) of the VUS-CNVs encompassed known disease-associated genes, with 55.6% (5/9) located in dosage-sensitive regions defined by ClinGen. Notably, the *22q11.21* duplication (Case 6) lies within the DiGeorge syndrome critical region, the *17q11.2* duplication (Case 2) includes the *NF1* gene associated with neurofibromatosis type 1 (OMIM:613113), and the *14q22.1* deletion (Case 1) involves *BMP4*, a key gene in cardiac development (OMIM:112262).

In our cohort of nine VUS-CNVs, variant size and genomic content appeared to correlate with phenotypic complexity. All VUS-CNVs in cases with isolated CHM were small (0.37–1.07 Mb), whereas those associated with extracardiac structural anomalies or complex CHM were larger (2.13–26.0 Mb), including two variants exceeding 20 Mb. Notably, VUS-CNVs ≤2 Mb were observed only in isolated CHM, while all variants >2 Mb occurred in non-isolated phenotypes.With respect to genomic burden, VUS-CNVs in the extracardiac anomaly group contained more OMIM-listed disease-associated genes (mean: 5.2) compared to those in the isolated CHM group (mean: 1.5). Among the four VUS-CNVs harboring three or more such genes, three were associated with extracardiac anomalies, whereas only one was seen in an isolated CHM case.Given the limited number of VUS cases in each subgroup (*n* ≤ 3), these findings represent descriptive observations rather than statistically significant associations. Nevertheless, they are consistent with the general principle that larger CNVs involving multiple dosage-sensitive genes are more likely to underlie multisystem developmental disorders.

Notably, Cases 7 (*5q22.1* duplication) and 9 (21.2 Mb heterozygous deletion) exhibited significant clinical phenotypes despite the absence of clearly defined pathogenic genes in OMIM or ClinGen. Additionally, the 26 Mb deletion in Case 4, although of uncertain significance, encompasses multiple genes associated with recessive disorders, potentially increasing disease risk. Database comparison showed that 55.6% (5/9) of the VUS-CNVs had pathogenicity reports in ClinVar or DECIPHER, with 2 cases (Cases 7, 8) absent from the DGV population frequency database, suggesting potential clinical significance. All gene annotations were based on the OMIM database (version 2024.03, updated on 2025-12-25).

### 4.3 VUS-CNVs and prenatal counseling

Genetic diagnosis facilitates early identification of fetal CHMs, guides further evaluation in cases with additional extracardiac anomalies, provides prognostic information, aids in genetic counseling, and informs future family planning (18, 19). CMA can simultaneously detect numerous chromosomal abnormalities and submicroscopic chromosomal mosaicism across the entire genome, a capability confirmed by multiple studies (24–26), and has been applied to detect chromosomal abnormalities in prenatal and postnatal subjects with CHMs. Moreover, CMA can identify gene mutations associated with CHMs, uncovering additional clinically significant CNVs. This not only enables broader genetic testing for CHM patients but also determines the genetic etiology of birth defects and enhances awareness of other genetic risks. Further CMA testing in fetuses with cardiac ultrasound abnormalities provides valuable genetic information for genetic counseling, postnatal interventions, and clinical research.

Although CMA can detect CNVs as small as 50 kb, its application as a routine diagnostic tool faces challenges due to the abundance of CNVs in the normal population, most of which are benign or of uncertain significance (VUS-CNVs), lacking clinical description (27). Current clinical practice classifies CNV pathogenicity based on size, population frequency, known genotype-phenotype correlations, and protein-coding gene content. This classification directly impacts the interpretation of microarray data. Indeed, VUS-CNVs pose a significant challenge for prenatal genetic counseling due to their unknown penetrance and clinical impact. Studies indicate that VUS-CNVs are classified as such simply due to insufficient data to confirm pathogenicity (28). Therefore, further clinical analysis and research are needed to provide adequate prenatal genetic counseling and explore the potential pathogenicity of VUS-CNVs.

Fetal VUS-CNVs often introduce clinical uncertainty, which may lead parents and healthcare teams to opt for pregnancy termination under concerns about potential risks. Despite limited evidence of high pathogenicity, psychological stress, misinterpretation of results, and the lack of unified clinical management guidelines significantly increase the decision-making burden. Qualitative studies show that even with low actual pathogenic risk, the ambiguity of VUS-CNVs can cause prolonged parental anxiety, with some opting for termination due to an inability to tolerate uncertainty (4).In our cohort, three of nine pregnancies with VUS-CNVs were terminated: one due to severe tetralogy of Fallot (with VUS amplifying prognostic concerns), one due to multiple structural anomalies (cardiac, intestinal, and renal) where the VUS contributed to perceived genomic risk, and one driven primarily by anxiety about uncertain neurodevelopmental outcomes despite isolated cardiac findings. The remaining six pregnancies continued to term, and all live-born infants showed no major abnormalities during short-term follow-up.This pattern aligns with broader literature: a study of 94 fetuses with VUS-CNVs reported that only 6 continued pregnancy while 8 opted for termination (29), reflecting heightened caution in clinical practice. Counseling approaches also influence decisions—some clinicians adopt “option-oriented counseling,” explicitly discussing termination or continuation, while others use “preference-informed counseling,” focusing on family values and psychological resilience (30). These differences further highlight how VUS-CNVs, even without proven pathogenicity, can heavily sway reproductive choices.

However, emerging follow-up data suggest potential overestimation of risk. A long-term study of 139 VUS-CNVs found only 6.5% were later reclassified as pathogenic, and many born children lacked expected phenotypes (31). In our series, the majority of families chose to continue pregnancy, and postnatal outcomes were reassuring—underscoring that termination decisions based solely on VUS-CNVs warrant careful multidisciplinary counseling, with emphasis on phenotypic context, variant characteristics, and psychological support.

Regarding accuracy, CMA is a highly accurate first-tier clinical technique but is costly. Currently, next-generation sequencing (NGS)-based CNV-seq is emerging as an alternative to CMA for detecting clinically significant chromosomal abnormalities. From a health economics perspective, the future trend in prenatal genetic testing may involve combining CNV-seq and whole-exome sequencing, enabling simultaneous detection of CNVs and monogenic disorders in a single assay (32). Our findings suggest that microdeletion/duplication syndromes and monogenic diseases may coexist, and CNVs may help uncover recessive conditions. Therefore, integrated genomic assessment may hold significant value in prenatal diagnosis. Thus, it can be inferred from this study that CNV-seq may become the primary technical strategy for prenatal diagnosis of known genetic disorders in cases with fetal structural anomalies.

## 5 Limitations and future perspectives

This study is limited by a small sample size (*n* = 9), which restricts the statistical power and generalizability of the observed associations between VUS-CNVs-CNV characteristics and phenotypic subgroups. Moreover, parental testing to determine the inheritance pattern of VUS-CNVs-CNVs was not performed in all cases, hindering accurate interpretation of pathogenicity and recurrence risk. In addition, the analysis was confined to copy number variants detectable by CMA and did not explore potential contributions from single nucleotide variants, small insertions/deletions (indels), or other genetic or epigenetic factors that may underlie congenital heart malformations (CHMs), particularly in isolated cases. Future studies should focus on larger prospective cohorts with systematic phenotyping and routine parental CMA testing to validate these findings. Long-term clinical follow-up of children born with VUS-CNVs-CNVs is crucial for establishing reliable genotype-phenotype correlations and ultimately clarifying their clinical significance in prenatal counseling.

## 6 Conclusion

Although variants of uncertain significance (VUS-CNVs) are uncommon in fetuses with congenital heart malformations (CHMs), they occur more frequently in non-isolated phenotypes and carry the potential for future reclassification—posing significant challenges in prenatal genetic counseling. These two aspects underscore the necessity of trio-based genomic testing, cautious interpretation of results, and longitudinal follow-up. Given the limited sample size of this study, no causal relationship between VUS-CNVs and clinical phenotypes can be inferred; however, our findings support a risk-stratified management framework that integrates detailed fetal phenotyping, trio analysis, and postnatal follow-up to enable accurate variant interpretation and informed decision-making.

## Data Availability

The datasets presented in this study can be found in online repositories. The names of the repository/repositories and accession number(s) can be found in the article/Supplementary Material.
